# 

**Published:** 1897-08

**Authors:** 


					﻿Vol. XVII.
AUGUST, -1897.
NO. 8.
THE
HOMEOPATHIC pH^lClAJI,
A Monthly Journal of Homoeopathic Materia Medica
and Clinical Medicine.
“ He is a freeman whom truth makes free, and all are slaves beside."
"Seek the Truth; come whence it may, cost what it will."
EDITED AND PUBLISHED BY
WALTER M. JAMES, M. D.
PHILADELPHIA :
TTq. 1231 LOCUST STREET.
LONDON :
ALFRED HEATH & CO., Sole Agents toe Great Britain,
114 Ebury Street, Eaton Square.
Yearly Subscription, $2.50, invariably in advance. Single numbers, 25 etc.
Entered at the Philadelphia Poat-Office aa Second-Claaa Mall Matter.
				

